# Health service availability and health seeking behaviour in resource poor settings: evidence from Mozambique

**DOI:** 10.1186/s13561-015-0062-6

**Published:** 2015-09-02

**Authors:** Laura Anselmi, Mylène Lagarde, Kara Hanson

**Affiliations:** 1 Manchester Centre For Health Economics, Institute of Population Health, The University of Manchester, Jean McFarlane Building, Oxford Road, Manchester, M13 9PL UK; 2 Department of Health Service Research, London School of Hygiene and Tropical Medicine, London, UK; 3 Department of Global Health and Development, London School of Hygiene and Tropical Medicine, London, UK

**Keywords:** Health seeking behaviour, Demand for health care, Health care availability, Reverse causality, Instrumental variables, Mozambique

## Abstract

Low-income countries are plagued by a high burden of preventable and curable disease as well as unmet need for healthcare, but detailed microeconomic evidence on the relationship between supply-side factors and service use is limited. Causality has rarely been assessed due to the challenges posed by the endogeneity of health service supply.

In this study, using data from Mozambique, we investigate the effect of healthcare service availability, measured as the type of health facilities and their level of staffing and equipment, on the individual decision to seek care. We apply an instrumental variable approach to test for causality in the effect of staff and equipment availability on the decision to seek care and we explore heterogeneous effects based on the distance of households to the closest health facility.

We find that living in the proximity of a health facility increases the probability of seeking care. A greater availability of referral health services in the locality has no significant effect on decision to seek care, while greater availability of staff and equipment increases the probability of seeking care when ill. Demand side barriers to health care use exist, but have a smaller impact when health care services are available within one hour walking distance.

## Introduction

Despite the high burden of preventable and curable disease in Low and Middle-Income Countries (LMICs) [1], there is considerable unmet need for health care [2]. Service availability is still limited and numerous barriers to access exist [3], preventing service use especially for the poorer socio-economic groups [4, 5]. Given these premises, exploring the determinants of service utilisation is central to identifying the causes of inequalities in health and health care. Especially where service provision is constrained and unequal across geographic areas, quantifying the causal effect of health care supply on use is key to understand inequity in all dimensions of health care financing and provision. Identifying the separate effects of supply and demand-side determinants of health care use may provide indications to policy makers about the most effective levers to increase access and encourage the use of services when needed.

The empirical literature on the determinants of health care use in LMICs has mostly relied on household survey data. Health seeking behaviour has been analysed by estimating the individual probability of seeking care when ill, or the probability of choosing a specific type of provider [6–11]. Due to the limited data on health care services available to the household, most studies have focused on the influence of demand-side factors, including individual demographic and socio-economic characteristics, as well as the indirect cost of using services, proxied by the travel time to the nearest health facility (HF). This body of literature highlights the existence of demand-side barriers to service use such as household geographic remoteness (and therefore difficulties in reaching the providers), low education levels, cultural aspects and poor economic conditions.

A limited number of quantitative empirical studies have analysed how aspects of access to or quality of health services supply affect the demand for health care [8–11]. The existing studies have so far captured the effects of availability and affordability, two key dimensions of access to health care [12]. Affordability has been proxied by user fees [8, 13], while availability has been measured through structural indicators, such as the number, type and conditions of health infrastructure [9] and the availability in HFs of staff [10], equipment [14] and drugs [9, 11]. Fewer studies have analysed the impact of quality on service use, using measures of technical quality, such as staff adherence to the treatment protocol [10, 15], or patients’ perceptions of quality [16]. Although HF characteristics have been interpreted as proxies for structural quality, it could be argued that they are capturing health care availability through the HFs’ capacity for service provision. The evidence points to the existence of a positive correlation between the availability and quality of health services and their use [9, 10, 14].

Most of the existing studies assess the correlation between supply and use of health care services but they are not able to determine the causal impact of supply factors on health care use due to potential reverse causality. Indeed more and better resources for service provision are likely to be allocated where use is higher. Although the endogeneity of health care services supply has been acknowledged in the literature [17, 18], the issue has been addressed in only one of the studies looking at the determinants of health care seeking in LMICs. Kumar et al. (2014) [19] used an instrumental variable (IV) approach to estimate the causal impact of the household distance to the HF (interpreted there as a measure of access) on institutional deliveries.

In this study, we seek to investigate the effect of health service availability on the decision to use health care when ill. We use household survey and routine HF data from Mozambique and measure health service availability along two dimensions: the type of HF available in the locality where a household lives (i.e. lower-level HFs providing only basic primary care vs. higher-level HFs providing extended primary and secondary care) and their resources in terms of staff and equipment, as a proxy for their capacity for effective provision of care. We consider the number and type of HFs to be fixed and exogenous in the period covered in this study, which would not allow for adjustment of health care supply to utilization patterns. However, since staff and equipment could be allocated to HFs where service use is higher there is an endogeneity problem which we overcome by following Kumar et al. (2014) [19] and using an instrumental variable approach. Specifically, we use the availability of housing for personnel as an instrument for staff and equipment. Finally, we explore heterogeneous effects by carrying out the analysis separately on the sub-samples of individuals living close (less than one hour walking time) and far (more than one hour walking time) from the nearest HF.

This study contributes to the existing literature in two ways. First, it adds to the limited evidence on the effect of health care supply on health seeking behaviour in LMIC, and in particular in Mozambique. Second, we test for causality in the relationship between health service availability and use through an instrumental variable approach. The next sections describe the study setting and the data, present the methods and the results and discuss and conclude.

## Setting and data

### Country setting

Mozambique is a sub-Saharan country with a population of 26 million. After independence from Portugal in 1975 and a subsequent civil war which lasted until 1992, the country has experienced peace, sustained economic growth over 6 % per annum and improvements in socio-economic indicators. For example, between 1997 and 2007, the GDP rose from 236 to 454 USD per-capita, the share of population living below the poverty line felt from 69 % to 55 % and the primary school completion rate increased from 22 % to 77 % (MPD, 2010).

Health care provision progressively expanded and health indicators improved. The number of health facilities increased from 1,210 in 2004 to 1,392 in 2011 and the number of doctors from 424 in 2000 to 1106 in 2010. Infant and under-five mortality reduced from 106 to 64 per thousand live-births and from 158 to 97 per thousand live-births between 1996 and 2011 [20]. However, life expectancy is still low (49 years), and similarly to other LMICs, the burden of disease is still high and predominantly constituted by preventable and curable diseases, most notably HIV/AIDS, malaria and respiratory diseases (IHME, 2013), which are also among the major causes of under-five mortality [21]. Inequalities in health and health care use still exist, and appear to be related to household socio-economic indicators (mainly wealth and education) and inequalities in health care availability across provinces and districts [22–25].

Health care is mostly publicly funded and provided. Central, provincial and district levels constitute the backbone of the top-down hierarchical health sector organization. Specialised care is managed at provincial level and provided by central or provincial hospitals, while primary and secondary care are managed at district level and provided by district hospitals, health centres and clinics. Clinics provide only basic primary care, health centres provide inpatient and general medicine consultation, while district hospitals provide also small surgery. At least one health centre is available in most districts particularly where a district hospital is not. During the time period covered by the data used in this analysis private care was limited to a few clinics, mainly concentrated in the capital [23].

Inequalities in the supply of health services in Mozambique are a reflection of disparities across provinces, districts and localities in the number and type of HF, as well as of human, financial and physical resources, such as drugs, consumables and equipment [22, 25, 26]. National directives from the MoH set the classification of HFs as well as, for each type of HF, norms for the minimum staff and equipment requirements based on the minimum inputs required to deliver the health care that a HF ought to provide to the population in the catchment area [27]. To improve recruitment and retention of professional health care workers outside the capital city Maputo, houses should be made available to mid- and high-level staff next to the HFs where they are working [28]. Despite these existing norms, lack of adequate equipment, staff and housing options are widespread across HFs in the country, and much more prevalent in some provinces and districts than others [25, 26].

User fees in public HFs are low: in local currency, Mozambican Meticais (MZM) 2 and MZM 1 for outpatient consultation in urban and rural areas, MZM 5 for all drug prescriptions and MZM 10 for inpatient care (equivalent to USD 0.07, USD 0.04, USD 0.16 and USD 0.32 respectively) and exemptions cover the large majority of the population [23]. Although higher fees are applied to prevent unreferred access to district and provincial hospitals (known as “bypassing”), anecdotal evidence of unreferred cased in district hospitals and provincial hospitals exist. Indeed, when individuals have access and can afford user fees they may decide to go directly to hospitals which offer a wider range of services and have extended working hours [23]. Beyond the limited direct costs implied by user fees, the indirect cost implied by distance from the HF and low levels of education and income limit the use of health care services [6, 7]. Provincially and temporally limited evidence suggests that the presence of trained health personnel in HF may influence institutional deliveries but not the use of outpatient care [14].

### Data

In this study, we use data on health care utilisation and on individual, household and community characteristics from the 2008/2009 household budget survey [29]. The sample consists of 10,831 households and 51,188 individual observations (9,362 households and 45,356 individuals in 847 communities excluding Maputo City) and is representative at provincial and urban and rural areas level. Data were collected through household and community questionnaires administered in the 599 rural communities. As in similar surveys, information on health care seeking (decision to seek care and choice of provider) is available only for individuals who reported illness in the past two weeks. Following the approach adopted in most of the existing literature, we restrict the sample of the analysis to those individuals who reported illness in the recall period. Measures of household (real) consumption per capita, spatially and temporally adjusted, were calculated by the Ministry of Planning and Development (MPD) for the third national poverty assessment, based on the Household Budget Survey 2008/2009 data [30, 31]. Adult equivalence scales were also provided by the MPD [32]. Data on HFs are derived from the National Health Information System [33] as provided by the Ministry of Health (MoH) in June 2012. A complete list of existing HFs is available for 2009, with information on staffing, equipment and housing for personnel. We verified the existence of each HF and its location based on a census of HFs undertaken in 2007 [34] and resolved mismatches through consultation with the relevant provincial or district directorates of health. Since routine data collected at local level may be biased and resource availability may be understated in less resourced HFs, to minimize inconsistency and bias, we cross-checked information on availability of staff and equipment across all available years (2008, 2009, 2010 and 2011). When a large discrepancy was found, the 2009 value was substituted with the average across the four years, to avoid using data that reflect availability in an exceptional period rather than a typical one. If the discrepancy was found for one year only, the exceptional year was excluded from the calculation if the average. A total number of 1,261 HFs providing primary and secondary care constituted the database in 2009. Data for the HF located in four districts (Mecula, Ibo, Tambara, Massingira), where less than one percent of the national population live, were not available and the districts were excluded from the analysis.

Information about the recommended minimum service coverage, staffing, equipment and availability of staff housing for each type of HF were extracted from official documents [24, 27, 28] and used to calculate for each item the ratio of the actual availability and the minimum set by norms. The norms used as a benchmark for the items included in the HF routine data are the following (reported for clinics, health centre and district hospitals respectively): health workers with basic level health training: 6, 13, 39; medium level health training: 1, 9, 29; high level health training: 0, 1, 9; and availability of an autoclave: 1, 1,1; bike: 2, 2, 2; motorbike: 0,1,9; and houses for staff: 0,1,9.

Since the Household Budget Survey does not provide information on the specific HF visited by individuals, we merged household survey and HF data at the locality level. In 2009, excluding Maputo City, the country was organised in 10 provinces, 142 district administrations and 1272 localities. Districts comprise between 1 and 22 localities, which cover a population between 250 and 50,000 people, except for some urban localities which cover up to 150,000 people. The organization of the public health sector referral system and the limited presence of alternative care providers led us to focus on the decision to visit a public HF providing outpatient care when ill. We assume that individuals visit the closest HF. Since most HF catchment areas fall within the administrative boundaries of the locality of residence, we assume that the closest HF is within the locality of residence of the household.

Because of their unusual pattern of health service provision and peculiar demographic and socio-economic characteristics compared with the rest of the country, we excluded Maputo City and Matola from the analysis

## Methods

### Estimating the probability of seeking care

The economic analysis of health care use has so far been rooted in a random utility model framework [35, 36], where individuals maximize their utility according to preferences over health and the consumption of other goods, conditional to their budget constraint, which incorporates individual income and the prices of consumables. The individual utility function can therefore be written as:

1 $$ {U}_i=\left({H}_i, {Z}_i\ \right) $$

where *H*_*i*_ is health status, which depends on the decision to seek care, and *Z*_*i*_ is the bundle of other goods consumed by individual *i*.

Individuals derive indirect utility from health care through the improvement of their health status and they choose from the affordable combinations of health care and other consumables the one that maximizes their utility:

2 $$ {U}_i^{*}= \max\ \left({U}_i^{S1},{U}_i^{S0}\right) $$

where ${U}_i^{S1}$ and ${U}_i^{S0}$ are the utility levels associated with using health care or not.

Since both *H*_*j*_ and *Z*_*j*_ depend on a set of individual, household and community characteristics, including health care use, using a latent variable approach, we defined the observed decision to seek care as a function of the observed determinants of health care demand and supply:

3 $$ \begin{array}{c}{y}_{icl}^{*}={U}_i^{S1}-{U}_i^{S0}={\alpha}_1+{\beta}_1\ {X}_{icl}+{\beta}_2{D}_{cl}+{\beta}_3\ HL{F}_l+{\beta}_4HR{E}_l + {\upvarepsilon}_{\mathrm{icl}}\\ {}{S}_{icl}=\left\{\begin{array}{c}\hfill 1\  if\ {y}_{icl}^{*}\ge\ 0\hfill \\ {}\hfill 0\  if\ {y}_{icl}^{*} < 0\ \hfill \end{array}\right.\end{array} $$

where ${y}_{icl}^{*}$ is the unobserved difference between the utility from seeking (${U}_i^{S1}$) and not seeking care (${U}_i^{S0}$), and *S*_*icl*_ is a dummy taking value 1 if the individual *i*, in community *c* and locality *l*, is better off when seeking care from a public provider, and 0 vice versa. Due to the lack of additional information we assume that each episode of health care seeking is an initial contact and we do not consider follow-up visits.

*HLF*_*l*_ and *HRE*_*l*_, capture the supply-side characteristics in locality *l*, where we assume that the HF closest to the household is located. *HLF*_*l*_ is the proportion of higher level HFs (health centre and district hospital) out of all HFs in locality *l* and accounts for the type of service which is accessible. *HRE*_*l*_ is an index of HF staffing and equipment, to account for technical quality of the service provided. *HRE*_*l*_ is measured as the ratio of available to minimum staff and equipment required by norms averaged across the following six dimensions: basic, medium and high level trained health cadres hired by the government, functional motorbike, car and autoclave. To capture the complementarities between human resources and equipment inputs in service delivery, we attributed the same weight to all dimensions. For localities with more than one HF, we calculated *HRE*_*l*_ by averaging across HFs and based on the assumption that individuals would visit the closest HF in the same district we imputed *HLF*_*l*_ and *HRE*_*l*_ using the equivalent average figures at district level for localities without HF. User fees are not included here as supply-side determinant of service use since they are minimal and we do not expect variability across HFs.

*D*_*cl*_ includes a set of dummies for the time required to walk between the community and the closest HF. Three dummies are defined according to the following thresholds: 0–59 minutes, 60–119 minutes and 120 minutes or more, which we used as the reference category. Distance from the closest HF was set to 0–59 minutes for households in urban areas to which the community questionnaire did not apply but where HFs are more concentrated. Distance from the HF, as well as transport availability and employment conditions, defined below, capture the indirect costs of using health care.

*X*_*icl*_ is a vector of additional individual, household and community characteristics:

- Gender and age to account for specific health care needs;
- Two non-mutually exclusive dummies for self-defined employment to account for the monetary and time opportunity cost of taking time off to visit a HF: permanently employed (versus seasonally or occasionally) and non-remunerated housekeeping worker.
- The highest level of education attained among household members, measured by years of schooling, as a proxy of social status, was preferred to the commonly used level of education of the head of household who in the specific context often had limited schooling opportunities due to civil war disruptions;
- The household adult equivalent consumption per capita, log-transformed to allow for nonlinear effects, to capture the economic condition;
- The average number of household members per room, to capture the availability of assets which is not captured by and may diverge from consumption measures of economic status [37];
- The availability of a latrine in the house, to account for household access to sanitation;
- The availability of public transport reaching the community, to account for geographic remoteness and ease of travelling to, and accessing, a HF;
- The month of the interview, corresponding to the month of the reported illness, to account for disease seasonality.

From the empirical specification shown in (3) we estimated the probability of seeking care, using a probit model:

4 $$ \Pr \left({S}_{icl}=1\Big|{X}_{icl},\ {D}_{cl},\ HL{F}_l,\ HR{E}_l\right)=\Phi \left({\alpha}_1+{\beta}_1\ {X}_{icl}+{\beta}_2{D}_{cl}+{\beta}_3\ HL{F}_l+{\beta}_4HR{E}_l\right) $$

We corrected for clustering at the locality level, the lower administrative level which incorporates villages with similar characteristics in terms of health care and other public service provision, and we included dummies for province fixed effects. The analysis was carried out using Stata 13.

### Using an instrumental variable to test for causality in the effect of health services availability

We use an instrumental variable (IV) approach to test for causality in the relationship between health care availability and the decision to seek care.

The availability at the HF of staff houses in good physical condition was selected as an instrument for the availability of staff and equipment. Since it has no effect on the individual decision to seek care other than through the availability of staff and equipment, for which is it is a good predictor, the availability of staff houses satisfies both the relevance and external validity conditions [38].

Indeed, staff housing is an important non-financial benefit for the retention of human resources in rural areas [39]. In Mozambique, according to a recent study, after salary, the availability of housing is the most important incentive for health workers to accept a placement outside of the capital [40]. Housing for personnel is therefore likely to increase health care personnel in the HF, and in turn equipment and other resources, such as drugs [41], which likely depend on the presence of HF staff.

Furthermore, since 2007, districts have been given autonomy to build clinics and staff housing, and started at the same time to receive decentralised financial resources from the Ministry of Finance for small local initiatives, for which the prioritization criteria were not fixed. Decisions on allocations would ultimately depend on the quality of the investment proposal. New houses for personnel were frequently built and old houses refurbished, given the relatively small investment required and the potential gain from attracting extra health staff [23]. Because of the decentralization of responsibilities and financial resources , the distribution of staff housing does not depend exclusively on provincial or national health administrations (MISAU, 2012c) and is therefore not potentially correlated with the patterns of service use.

We estimated an IV probit model which includes a set of two equations: the first estimates the probability of seeking care, as previously described in equation (3), and the second predicts HF staff and as a function of housing for personnel as well as of the other independent variables:

5 $$ \begin{array}{c}{y}_{icl}^{*}={\alpha}_1+{\beta}_1\ {X}_{icl}+{\beta}_2{D}_{cl}+{\beta}_3\ HL{F}_l+{\beta}_4HR{E}_l + {\upvarepsilon}_{\mathrm{icl}}\\ {}HR{E}_l={\gamma}_0+{\gamma}_1\ {X}_{icl}+{\gamma}_2{D}_{cl}+{\gamma}_3\ HL{F}_l + {\gamma}_4\ H{S}_l+{v}_{icl}\\ {}{S}_{icl}=\left\{\begin{array}{c}\hfill 1\  if\ {y}_{icl}^{*}\ge\ 0\hfill \\ {}\hfill 0\  if\ {y}_{icl}^{*} < 0\ \hfill \end{array}\right.\end{array} $$

As in (3), *S*_*icl*_ is a dummy taking value 1 if the individual *i*, seeks care from any public provider and 0 vice versa, *HLF*_*l*_ and *HRE*_*l*_ capture supply-side characteristics, D_cl_ is the set of dummies capturing the distance from the closest HF and *X*_*icl*_ is a vector including other demand-side characteristics and controls, as in (3). *HS*_*l*_ is the ratio of available staff housing to the minimum set by norms for each type of HF. In localities where more than one HF is available, we average housing availability across HFs and in localities where no HF is available, we inpute the average across all district HFs.

After estimating the model, we checked for the endogeneity of ${HRE}_l$ by looking at the Wald test of exogeneity, which in the case of a single endogenous variable reduces to testing the null hypothesis of no correlation (rho) between the error terms of the first and second equations, ${\varepsilon}_{icl}$ and ${v}_{icl}$. A significant rho and a Wald test rejecting the null hypothesis of exogeneity would suggest endogeneity of HF staff and equipment [42]. A significant coefficient associated with *HS*_*l*_ (${\gamma}_4$) and an F statistic of the first-stage OLS regression greater than 10 would suggest that *HS*_*l*_ is a non-weak instrument [42].

### Exploring heterogeneity in the effect of health care availability

To explore the heterogeneity in the effect of health care availability, we estimated the effect of staff and equipment availability on the decision to seek care separately for the two-subsamples of households according to their proximity to a HF. Households are considered close to and far from a HF if it can be reached in less than one hour by foot and vice-versa.

## Results

Individuals reporting an illness during the two weeks preceding the interview represent 13 % of the whole survey sample. Of these, 61 % sought care from a public HF, while only 3 % sought care from other providers (see Table 1 for descriptive statistics). 92 % of all individuals and 97 % of those residing within one hour of the closest HF live in a locality with at least one HF. Around 80 % of the HFs are clinics and they have 77 % and 60 % of the minimum staff and equipment and staff housing set by norms. Greater variability is observed across localities, rather than across districts, and the mean household characteristics appear to be an average between the 60 % of the population living within one hour walking distance from a HF and the remaining 40 %.

**Table 1:** Descriptive statistics for individuals ill in the two weeks prior to the interview, Mozambique 2009

Variable	Mean	SD	Min	Max
Service utilisation				
Number of visits to HF (previous month)	0.75	0.83	0.00	15.00
Seeking care from a public HF	0.61	0.49	0.00	1.00
Seeking care from other providers	0.03	0.18	0.00	1.00
Demand-side characteristics				
Woman	0.58	0.49	0.00	1.00
Age	24	22	0	99
Highest level education in household (years schooling	5.44	3.23	0.00	18.00
Employed in permanent work	0.44	0.50	0.00	1.00
Employed in non remunerated housekeeping work	0.20	0.40	0.00	1.00
Household adult equivalent consumption per-capita (MZM per day)	33	33	1	921
Number of household members per room	1.98	1.23	0.03	10.00
Latrine in the house	0.58	0.49	0.00	1.00
Transport reaching the community	0.63	0.48	0.00	1.00
Urban	0.37	0.48	0.00	1.00
Supply-side characteristics				
1 hour time distance from closest HF	0.60	0.49	0.00	1.00
2 hours time distance from closest HF	0.08	0.28	0.00	1.00
More than 2 hours time distance from closest HF	0.31	0.46	0.00	1.00
HF in locality of residence	0.92	0.28	0.00	1.00
Percentage of HCs and DHs among HFs in locality	0.23	0.24	0.00	1.00
Percentage of HC s and DHs among HFs in district	0.18	0.11	0.33	1.00
HF staff and equipment index (locality average)	0.50	0.26	0.00	2.13
HF staff and equipment index (district average)	0.47	0.18	0.19	1.25
HF housing availability index (locality average)	0.60	0.89	0.00	10.00
HF housing availability index (district average)	0.60	0.62	0.00	3.38

Number of observations: 6,034

HC: health centre, DH: district hospital

Table 2 presents the results from the probit and IV probit models estimated on the whole sample. The probit model (Column 1) shows that living in proximity of a HF increases the probability of seeking care when ill, while neither the type of HF available in the locality nor their staff and equipment affect the decision to seek care. Among the demand side characteristics, as expected, having better education, income and assets, being permanently employed, living in a house with latrine and in a community reached by public transport, all increase the probability of seeking care when ill. Interestingly, being employed in unpaid housekeeping work and residing in urban area reduce the probability of seeking care from a public HF, which may reflect time constraints and greater availability of alternative providers.

**Table 2:** Determinants of the decision to seek care when ill, Mozambique 2009

	Probit	IV-Probit	
		2^nd^ stage	1^st^ stage
Supply-side characteristics			
Percentage of HCs and DHs among HFs in locality	0.042	0.033	−0.118
	(0.038)	(0.040)	(0.077)
HF staff and equipment index (locality average)	0.043	0.122	
	(0.029)	(0.083)	
HF availability of housing (locality average)			0.098***
			(0.023)
HF time distance: < 1 hour	0.184***	0.182***	0.016
	(0.026)	(0.026)	(0.019)
HF time distance: 1–2 hour	0.053	0.050	0.034
	(0.033)	(0.034)	(0.028)
Demand-side characteristics			
Woman	0.011	0.011	0.000
	(0.014)	(0.014)	(0.005)
Age	−0.005***	−0.005***	0.000
	(0.001)	(0.001)	(0.001)
Age squared	0.000***	0.000***	0.000
	(0.000)	(0.000)	(0.000)
Higher level education attained in household	0.012***	0.012***	0.005*
	(0.003)	(0.003)	(0.003)
Employed in permanent work	0.061***	0.063***	−0.015
	(0.020)	(0.020)	(0.013)
Employed in housekeeping work	−0.046**	−0.046***	0.002
	(0.020)	(0.019)	(0.009)
Log household consumption per-capita	0.032***	0.031***	0.014*
	(0.010)	(0.010)	(0.008)
Number of household members per room	−0.014**	−0.014**	−0.001
	(0.006)	(0.006)	(0.004)
Latrine in the house	0.029*	0.030*	−0.007
	(0.017)	(0.017)	(0.014)
Transport reaching the community	0.069***	0.068***	0.016
	(0.025)	(0.025)	(0.018)
Urban	−0.052**	−0.060**	0.138***
	(0.025)	(0.025)	(0.039)
Constant	Yes	Yes	Yes
Province dummies	Yes	Yes	Yes
Month dummies	Yes	Yes	Yes
Observations	6,026	6,026	
Pseudo R-Squared	0.090		
Log pseudolikelihood	−3676.406	−2916.161	
Rho			−0.054
Standard error			(0.056)
Wald test of exogeneity^(a)^			0.333
*p*-value			0.397
F-test first stage^(a)^			18.697***

Average marginal effects reported

1^st^ and 2^nd^ Stage refer respectively to the reduced-form and structural equations

Standard errors corrected for intra-cluster correlation at locality level in parentheses

^(a)^Adjusted for clusters (*N* = 452)

*HC* health Centre, *DH* district Hospital

***p < 0.01, **p < 0.05, *p < 0.1

The IV probit model (Columns 2 and 3) rules out the hypothesis of reverse causality bias in the probit estimates. Indeed the significance of the coefficient associated with the ratio of available to minimum number of houses in the first stage of the IV probit and the F-test (F = 18.7) confirm that it is a not a weak instrument. The Wald test (*p* = 0.397) result suggests that HF staff and equipment is not endogenous in the first place and the probit estimates are more efficient and should be preferred to the IV estimates [42]. The coefficient associated with HF staff and equipment in the probit estimates can be given causal interpretation. Even if we were to follow stricter criteria suggesting that the difference between the probit and IV probit coefficient associated with the availability of staff and equipment could per se be a sign of endogeneity [42] and therefore prefer the IV probit coefficient, we would still find a non-significant effect but a larger coefficient.

Table 3 shows the results of the probit models estimated on two sub-samples of individuals, living within one or more than one hour from the closest HF. Three findings emerge from the probit estimates on the subsample of individuals living close to a HF (Column 1). First, as before, the type of services provided in the locality has no significant effect on the decision to seek care. However, the availability of staff and equipment in local HFs has a positive and significant effect on the decision to seek care, with a marginal effect of 0.075. The latter corresponds to an increase of 0.00075 for each extra percentage point of the ratio of available to minimum HF staff and equipment. Since on average HF currently have only 50 % of the staff and equipment set by norms, reaching the minimum set by norms, on its own would increase the probability of seeking care for those who live near a HF by 0.04.

**Table 3:** Effect of supply-side characteristics on healthcare seeking according to distance from the closest health facility, Mozambique 2009

	Hhold lives within 1 hour from HF	Hhold lives more than 1 hour from HF
	Probit	Probit
Supply-side characteristics		
Percentage of clinics among HFs in locality	−0.025	−0.077
	(0.050)	(0.063)
HF Staff and Equipment index (locality average)	0.075***	−0.060
	(0.029)	(0.061)
HF time distance: 1–2 hour		0.054
		(0.036)
Demand-side characteristics		
Woman	0.026	−0.014
	(0.017)	(0.020)
Age	−0.004***	−0.007***
	(0.001)	(0.002)
Age squared	0.000**	0.000
	(0.000)	(0.000)
Highest level education in hhold (years schooling)	0.009***	0.018***
	(0.003)	(0.005)
Employed in permanent work	0.045**	0.085**
	(0.021)	(0.037)
Employed in housekeeping work	−0.046*	−0.038
	(0.028)	(0.027)
Log hhold consumption per-capita	0.028**	0.047**
	(0.011)	(0.020)
Number of hhold members per room	0.006	−0.039***
	(0.010)	(0.008)
Latrine in the house	0.034	0.017
	(0.024)	(0.026)
Transport reaching the community	0.106***	0.052
	(0.037)	(0.032)
Urban	−0.060**	
	(0.025)	
Constant	Yes	Yes
Province dummies	Yes	Yes
Month dummies	Yes	Yes
Observations	3,597	2,429
Pseudo R-squared	0.040	0.070
Log pseudolikelihood	−2,079.49	−1,557.05

Average marginal effects reported

Standard errors corrected for intra-cluster correlation at locality level in parentheses

Number of clusters: 1 hour walking distance: 234, more than 1 hour walking distance: 281

*HC* health Centre, *DH* district Hospital

***p < 0.01, **p < 0.05, *p < 0.1

The probit results for the population living further away from HFs (Column 4) show that neither the type of services available in the locality, nor the availability of staff and equipment have a significant effect on the decision to seek care. Unlike in the analysis performed on the whole sample, employment in housekeeping work, the availability of a latrine in the house and of public transport reaching the community have no significant effect for those living faraway from a HF. The non-significant effect of transport availability in the sub-sample considered here suggests that if the HF is further away the opportunity cost to reach it may be so high that travelling to it would not be considered an option.

### Robustness checks

We carried out five additional analyses to test the robustness of our results to the assumptions made and the methods chosen. The marginal effects associated with the two measures of availability of health services used throughout this paper, obtained by re-estimating all models for each robustness check, are summarized in Tables 4 and 5.

**Table 4:** Summary of robustness checks results for health care availability: average marginal effects associated with the percentage of clinics among HF in a locality or district, Mozambique 2009

	Whole sample	Hhold lives within 1 hour from HF	Hhold lives more than 1 hour from HF
	Probit	Probit	Probit
Main model			
Percentage of HC and DH among HFs (in locality)	0.042	0.025	0.077
	(0.038)	(0.050)	(0.063)
Percentage of HC and DH among HFs (in district)	0.57*	0.137	0.272
	(0.085)	(0.092)	(0.222)
Excluding provincial capitals			
Percentage of HC and DH among HFs (in locality)	0.037	0.011	0.077
	(0.042)	(0.060)	(0.063)
Percentage of HC and DH among HFs (in district)	0.236*	0.243*	0.272
	(0.121)	(0.142)	(0.221)
Village cluster SE			
Percentage of HC and DH among HFs (in locality)	0.042	0.025	0.077
	(0.038)	(0.050)	(0.056)
Percentage of HC and DH among HFs (in district)	0.157*	0.137	0.272
	(0.092)	(0.098)	(0.209)
District cluster SE			
Percentage of HC and DH among HFs (in locality)	0.042	0.025	0.077
	(0.038)	(0.055)	(0.061)
Percentage of HC and DH among HFs (in district)	0.157**	0.137	0.272*
	(0.081)	(0.100)	(0.150)

Average marginal effects reported

Standard errors corrected for intra-cluster correlation at locality level in parentheses when not differently specified

Constant, HFs Staff and Equipment, Demand-side characteristics, Province and month controls included

*HC* health Centre, *DH* district Hospital

***p < 0.01, **p < 0.05, *p < 0.1

**Table 5:** Summary of robustness checks results for health care availability: Probit and IV probit average marginal effects associated with HF staff and equipment in locality and district, Mozambique 2009

	Whole sample		Hhold lives within 1 hour from HF	Hhold lives more than 1 hour from HF	HF staff and equipment index endogeneity	Instrument strength
	Probit	IVProbit	Probit	Probit		
Main model						
HF staff and equipment index (locality average)	0.043	0.122	0.075***	−0.060	Exogenous	Strong
	(0.029)	(0.083)	(0.029)	(0.061)		
HF staff and equipment index (district average)	0.074*	0.464**	0.132***	−0.062	Exogenous except for all sample	Weak
	(0.042)	(0.230)	(0.044)	(0.087)		
Excluding provincial capitals						
HF staff and equipment index (locality average)	0.041	0.099	0.072**	−0.060	Exogenous	Strong
	(0.031)	(0.082)	(0.034)	(0.061)		
HF staff and equipment index (district average)	0.076	0.218*	0.155***	−0.062	Exogenous	Strong
	(0.049)	(0.117)	(0.059)	(0.087)		
PCA weighting						
HF staff and equipment index (locality average)	0.009	0.041	0.024**	−0.027	Exogenous	Strong
	(0.010)	(0.028)	(0.010)	(0.020)		
HF staff and equipment index (district average)	0.020	0.191*	0.041***	−0.022	Exogenous except for whole sample	Weak
	(0.013)	(0.109)	(0.014)	(0.024)		
Village cluster SE						
HF staff and equipment index (locality average)	0.043	0.122	0.075**	−0.060	Exogenous	Strong
	(0.033)	(0.087)	(0.037)	(0.059)		
HF staff and equipment index (district average)	0.074	0.464**	0.132**	−0.062	Exogenous except for whole sample	Weak
	(0.053)	(0.219)	(0.059)	(0.091)		
District cluster SE						
HF staff and equipment index (locality average)	0.043	0.122	0.075**	−0.060	Exogenous	Strong
	(0.029)	(0.083)	(0.030)	(0.057)		
HF staff and equipment index (district average)	0.074*	0.464*	0.132***	−0.062	Exogenous except for whole sample	Weak
	(0.043)	(0.245)	(0.047)	(0.081)		

1^st^ and 2^nd^ Stage refer to reduced-form and structural equation

Average marginal effects reported

Standard errors corrected for intra-cluster correlation at locality level in parentheses when not differently specified

Constant, Percentage of clinics among HFs, Demand-side characterictics, Province and month controls included

Exogenous if Wald test for exogeneity does not reject the null hypothesis of exogeneity at 5 % level

*HC* health Centre, *DH* district Hospital

***p < 0.01, **p < 0.05, *p < 0.1

First, since in settings with a number of different providers individuals may not automatically seek care from the closer one, but rather seek higher quality of care [43], we allow for individuals to seek care from a HF in their district rather than locality of residence and used district measures of health care availability. The coefficient associated with the availability of staff and equipment in these models is still significant and larger. However, the Wald test indicates endogeneity and housing for personnel is a weak instrument in this model, indicating that caution should be taken in inferring a causal relationship at district level. The level of service availability in a district has a positive and significant effect on care seeking.

Second, since in provincial capitals health care provision is notoriously more heterogeneous and housing allowances may be given to staff instead of providing accommodation, we excluded the provincial capital districts from the sample. The marginal effect of district average HF staff and equipment on the decision to seek care for individuals living in proximity of a HF is still positive, and even larger, and the proportion of HFs offering a higher level of services has a positive and significant effect for the whole sample, as well as for those living in proximity of a HF. In this specification the Wald test confirms exogeneity of the availability of staff and equipment.

Third, we used an alternative HF staff and equipment measure where we weight each of the six dimension using factor scores obtained from a principal component analysis (PCA). PCA is a multivariate statistical technique often used to reduce multiple dimensions into a unique indicator by weighting each of them proportionally to how much of the data variation they explain [44]. From the first principal component we take the following weights for the dimensions included: motorbike 0.4601, sterilizer 0.1888, car 0.4052, high level trained cadres 0.0173, medium level trained cadres 0.5054, basic level trained cadres 0.5769. Results confirm previous findings. However, the average marginal effect of HF staff and equipment is now smaller suggesting that the estimated impact is sensitive to the measures of health care availability and their combination. Nevertheless, lacking precise information on the relative weight of each dimension of health care availability there is no reason to believe that the relevance of each dimension of health care availability in explaining sample variability in the observed HF staff and equipment, would reflect their relevance for health care delivery.

Fourth, following the common practice [45], we corrected for clustering of standard errors at the lowest (village) and highest (district) levels to account for the Household Budget Survey design and the hierarchal organization of service provision, and found no relevant changes in results.

Fifth, we tested for the consistency of the effect across economic status, by including in the main model specification the interaction terms between the measures of health service availability and two dummy indicators for the poorest or richest income quintile and found no significant difference.

## Discussion

In this study, we set out to analyse the effect of health service availability, measured as the proportion of HFs offering inpatient and secondary care and the availability of staff and equipment in all HFs in the locality where the household resides, on individual health-seeking behaviour. We tested for causality in the effect of staff and equipment availability on the decision to seek care and we explored heterogeneous effects based on the distance of households to the closest health facility. We find that living in the proximity of a HF increases the probability of seeking care by 0.18. A greater availability of referral health services in the locality has no significant effect on the decision to seek care, while the availability of staff and equipment in all HFs, although small, has a positive and causal effect on the decision to seek care, but only among those individuals who can reach a HF within one hour. For them, an increase of 13 percentage points in the ratio of the available to minimum staff and equipment may lead to an increase of at least one percentage point in the probability of seeking care when ill. The lack of significance of breadth of services at the locality level may partly be due to a lack of variation in services provided at such a small level. However, the positive effect of a broader range of health services in a district is in line with anecdotal evidence of bypassing the referral system (i.e. visiting a higher level facility directly, without visiting a primary care facility first) to visit a HF providing a wider range of services and therefore better staffed and equipped in absolute terms [23].

For those living near a HF, the measure of health service availability that matters relates to the actual availability of inputs to provide the services, first because once distance is not a major barrier to use service, other factors may play a bigger role, and second because they probably have more and better information than those living further away. These findings are generally in line with previous studies from Mozambique [6, 7, 14]. Differences concern the insignificant effect of income [7] and of HF characteristics [14], which may be explained by the evolution of health seeking behavior over time and real increases in consumption per capita, respectively. Furthermore previous studies used data from a sub-sample of provinces and by the analysis of health behavior unconditional on illness reporting.

From a policy perspective, the results suggest that increased utilisation of health services can be achieved if service provision is scaled-up, which can be done through three different channels. First, health care services can be made more accessible to a larger population by increasing the number of HFs in a given area. Making a HF accessible within one hour walking distance would increase alone the probability of seeking care by 0.18 for the 40 % of the population that currently live further away. Second, the type of services provided in a given locality could be expanded, with existing HFs offering a wider range of health care services, or through a change in the ‘HF mix’ of a given area, with a greater proportion of HFs providing primary and secondary care. Third, governments could increase the availability of inputs necessary to make health services effectively available in a given HF (i.e. staff, equipment and drugs), for example to meet the minimum level set by official existing norms. Since on average HFs currently have only 50 % of the staff and equipment set by norms, reaching that standard would increase the probability of seeking care for those who live in the proximity of a HF by 4 %.

Previous studies suggested that making more resources available in existing HFs is more cost-effective than increasing the number of HFs in order to improve health care utilisation [17]. Our results suggest that choosing one policy or the other might have different equity implications, beyond cost effectiveness ones. Indeed, increasing the availability of staff and equipment would benefit the subsample of individuals living closer to HFs, who may correspond to the better-off groups of the population, but would not affect the probability of seeking care for those living further away. The latter would only increase if more HFs were made available where households live. Our results also suggest that there are demand-side barriers that have to be addressed to improve health care utilization among individuals needing care, namely income, education, and a type of employment. Differences across these dimensions seem to be more important determinants of the probability of seeking care when HF are not easily accessible suggesting that increasing the availability of health care may also potentially reduce inequalities in service use due to individual and household characteristics. Lowering the barriers to access related to distance would reduce the opportunity cost of visiting a HF, making it more affordable to individuals with lower socio-economic status.

As found by all previous studies on health care seeking behaviours in LMICs using data from surveys on living standards or similar, this study suffers from several limitations, mostly related to data availability. First, results are not representative for the entire population in the regional catchment area, but only for individuals who reported illness in the two weeks prior to the interview. However, illness reporting may be a reflection of self-perceived rather that objective measures of health. The presence of a possible systematic bias in health status self-assessment may translate into an over-estimation of the effect of the variables of interest because individuals who are more likely to use services (and who can more easily access a HF) are also more likely to report illness [46]. Second, the lack of information on health status and illness type and seriousness prevents a deeper understanding of the magnitude and heterogeneity of the effect associated with both demand and supply-side determinants of health care use. Third, we implicitly assume that individuals have a perfect knowledge of where HF are, what type of services they offer and how well staffed and equipped they are. If instead information was inversely correlated to distance from a HF, the lack of effect of supply-side determinants on the probability of seeking care could then be the reflection of lack of information. However, since the nature of the services provided in clinics and of the measure of their staff and equipment considered here is very basic and easily observable, it is likely that individuals get information before deciding whether to seek care. Fourth, the number and type, and therefore the distance from a HF, have been treated as exogenous in this analysis. However, while this assumption is plausible in the short run covered by the data, it is plausible that in the long run more HFs and providing a higher level of care may also be concentrated where individuals are more likely to use health care. Studies assuming a longer run perspective should therefore allow for endogeneity in HF availability and explore the complementarity/ substitution of decisions concerning the number, type and resourcing of HFs should be explicitly discussed. Finally, the analysis is limited to the first contact with a health care provider but does not explore the effect of demand and supply-side factors on the intensity of care received afterwards.

## Conclusion

In contexts where resources are limited, investing in service availability, in terms of number and type of HF as well as resources in HFs will contribute to better quality of care and encourage populations to use health services. However, while increasing the number of HF would increase the probability of seeking care we found that this effect would be small but significant only for those living closer to existing HFs. Such policies could benefit primarily more advantaged populations, while those in greater need would remain under-served without extending the services currently provided. Even when services are available, demand-side constraints still limit access to health care. However, geographic proximity of a HF reduces the opportunity cost and the relative effect of demand side factors and likely inequalities related to socio-economic factors.

Further research should aim to generate a deeper understanding of the relative importance and interplay between demand and supply-side determinants of service use and between the various dimensions of health care need, access and quality. The role of demand and supply-side factors should be explored not only in determining the decision to seek care, but also the choice of provider, the frequency of use and the effective use of more specialised services.

## References

[1] Lozano R (2012). Global and regional mortality from 235 causes of death for 20 age groups in 1990 and 2010: a systematic analysis for the Global Burden of Disease Study 2010. Lancet.

[2] Dupas P (2011). Health behaviour in developing countries. Annual Review of Economics.

[3] Ensor T, Cooper S (2004). Overcoming barriers to health service access: influencing the demand side. Health Policy Plan.

[4] Bonfrer I (2013). Does health care utilization match needs in Africa? Challenging conventional needs measurement. Health Policy Plan.

[5] Van de Poel E, Van Doorslaer E, O'Donnell O (2012). Measurement of inequity in health care with heterogeneous response of use to need. J Health Econ.

[6] Salvucci V (2014). Health provider choice and implicit rationing in healthcare: Evidence from Mozambique. Development Southern Africa Development Southern Africa.

[7] Lindelow M (2004). The utilization of curative health care in Mozambique: Does income matter?.

[8] Akin JS (1986). The demand for primary health-care services in the Bicol Region of the Philippines. Econ Dev Cult Chang.

[9] Akin JS, Guilkey DK, Denton EH (1995). Quality of services and demand for health care in Nigeria: a multinomial probit estimation. Soc Sci Med.

[10] Lepine A, Nestour AL (2013). The determinants of health care utilisation in rural Senegal. J Afr Econ.

[11] Mwabu G, Ainsworth M, Nyamete A (1993). Quality of medical care and choice of medical treatment in Kenya: an empirical analysis. J Hum Resour.

[12] McIntyre D, Thiede M, Birch S (2009). Access as a policy-relevant concept in low- and middle-income countries. Health Econ Policy Law.

[13] Heller PS (1982). A model of the demand for medical and health services in Peninsular Malaysia. Soc Sci Med.

[14] Lindelow M (2004). Understanding spatial variation in the utilization of health services: does quality matter?. Technical report.

[15] Leonard KL, Mliga GR, Haile Mariam D (2002). Bypassing health centres in Tanzania: revealed preferences for quality. J Afr Econ.

[16] Hanson K, Yip WC, Hsiao W (2004). The impact of quality on the demand for outpatient services in Cyprus. Health Econ.

[17] Collier P, Dercon S, Mackinnon J (2002). Density versus quality in health care provision: Using household data to make budgetary choices in Ethiopia. World Bank Econ Rev.

[18] Giuffrida A, Gravelle H (2001). Measuring performance in primary care: Econometric analysis and DEA. Appl Econ.

[19] Kumar S, Dansereau E, Murray C (2014). Does distance matter for institutional delivery in rural India?. Appl Econ.

[20] IHME (2013). Global Burden of disease profile: Mozambique.

[21] MISAU, INE, and ICF (2013). Moçambique Inquérito Demográfico e de Saúde 2011.

[22] Fernandes QF (2014). Effects of health-system strengthening on under-5, infant, and neonatal mortality: 11-year provincial-level time-series analyses in Mozambique. Lancet Glob Health.

[23] MISAU (2012). Revisão do sector Saúde 2007–2012.

[24] MISAU (2012). Proposta de manual para a alocação geográfica de recursos.

[25] MISAU and TARSC/EQUINET (2010). Equity watch: assessing progress towards equity in health in Mozambique.

[26] MISAU (2013). Informação estatística sumaria nacional (Analise de cinco anos: 2008–2012).

[27] MISAU (2002). Diploma Ministerial 127/2002, de 31 de Julho (Regulamento que define a caracterização técnica e enunciado das funções do Serviço Nacional de Saúde), BR n. 14 , Suplemento.

[28] MISAU (2007). Despacho do Ministério da Saúde num. 72 de 28 de Setembro de 2007 sobre o regulamento de atribuição de casas aos trabalhadores do Sector Saúde.

[29] INE (2010). Inquérito ao Orçamento Familiar 2008/2009.

[30] Arndt C, Simler K (2010). Estimating utility‐consistent poverty lines with applications to Egypt and Mozambique. Econ Dev Cult Chang.

[31] Arndt TC, Jones ES, Tarp F (2010). Poverty and wellbeing in Mozambique: Third national poverty assessment.

[32] MPF, UEM, and IFPRI (1998). Understanding Poverty and Well-Being in Mozambique: The First National Assessment (1996–97).

[33] MISAU (2012). Sistema de Informação de Saúde - Modulo Básico.

[34] MISAU (2007). Inventário nacional de infra-estruturas de saúde, serviços e recursos.

[35] Grossman M, Culyer and Newhouse (2000). The human capital model. Handbook of Health Economics.

[36] Becker G (1965). A theory of the allocation of time. Econ J.

[37] Lindelow M (2006). Sometimesmore equal than others: how health inequalities depend on the choice of welfare ndicator. Health Econ.

[38] Angrist JD, Pischke J-SE (2009). Mostly harmless econometrics: An empiricist's companion.

[39] Dolea C, Stormont L, Braichet J-M (2010). Evaluated strategies to increase attraction and retention of health workers in remote and rural areas. Bull World Health Organ.

[40] Honda A, Vio F. Incentives for non-physician health professionals to work in the rural and remote areas of Mozambique—a discrete choice experiment for eliciting job preferences. Hum Resour Health. 2015;13:23. 10.1186/s12960-015-0015-5 PMC4415227 25924927

[41] Wagenaar BH, et al. Stock-outs of essential health products in Mozambique – longitudinal analyses from 2011 to 2013. Trop Med Int Health. 2014;19(7):791–801. doi:10.1111/tmi.12314. Epub 2014 Apr 11. 10.1111/tmi.12314 PMC4479203 24724617

[42] Cameron AC, Trivedi PK (2009). Microeconometrics using Stata.

[43] Leonard KL (2014). Active patients in rural African health care: implications for research and policy. Health Policy Plan.

[44] Vyas S, Kumaranayake L (2006). Constructing socio-economic status indices: how to use principal component analysis. Health Policy Plan.

[45] Cameron AC, Miller DL. A practitioner’s guide to cluster-robust inference. J Hum Resour. 2015;50(2):317–372.

[46] Appleton S (1998). The impact of public services on health care and illness: A treatment effects model with sample selectivity. J Afr Econ.

